# Improvement of Bioactive Polyphenol Accumulation in Callus of *Salvia atropatana* Bunge

**DOI:** 10.3390/molecules29112626

**Published:** 2024-06-03

**Authors:** Izabela Grzegorczyk-Karolak, Wiktoria Ejsmont, Anna Karolina Kiss, Przemyslaw Tabaka, Wiktoria Starbała, Marta Krzemińska

**Affiliations:** 1 Department of Biology and Pharmaceutical Botany, Medical University of Lodz, 90-151 Lodz, Poland; wiktoria.ejsmont@stud.umed.lodz.pl (W.E.); wiktoria.starbala@stud.umed.lodz.pl (W.S.); marta.krzeminska@umed.lodz.pl (M.K.); 2 Department of Pharmaceutical Biology, Medical University of Warsaw, 02-097 Warsaw, Poland; anna.kiss@wum.edu.pl; 3 Institute of Electrical Power Engineering, Lodz University of Technology, 90-537 Lodz, Poland; przemyslaw.tabaka@p.lodz.pl

**Keywords:** abiotic stress, callus culture, LEDs, light spectrum, line selection, phenolic acids, rosmarinic acid

## Abstract

Callus cultures of the Iranian medicinal plant *Salvia atropatana* were initiated from three-week-old seedlings on Murashige and Skoog (MS) medium supplemented with α-naphthaleneacetic acid (NAA) and various cytokinins. Although all tested hormonal variants of the medium and explant enabled callus induction, the most promising growth was noted for *N*-(2-chloro-4-pyridyl)-*N*′-phenylurea (CPPU)-induced calli. Three lines obtained on this medium (cotyledon line-CL, hypocotyl line—HL, and root line—RL) were preselected for further studies. Phenolic compounds in the callus tissues were identified using UPLC–MS (ultra-performance liquid chromatography–mass spectrometry) and quantified with HPLC (high-performance liquid chromatography). All lines exhibited intensive growth and contained twelve phenolic acid derivatives, with rosmarinic acid predominating. The cotyledon-derived callus line displayed the highest growth index values and polyphenol content; this was exposed to different light-emitting diodes (LED) for improving biomass accumulation and secondary metabolite yield. Under LED treatments, all callus lines exhibited enhanced RA and total phenolic content compared to fluorescent light, with the highest levels observed for white (48.5–50.2 mg/g dry weight) and blue (51.4–53.9 mg/g dry weight) LEDs. The selected callus demonstrated strong antioxidant potential in vitro based on the 2,2′-azino-bis(3-ethylbenzthiazoline-6-sulfonic acid) (ABTS), 2,2-diphenyl-1-picrylhydrazyl (DPPH), and ferric reducing antioxidant power (FRAP) tests. Our findings confirm that the *S. atropatana* callus system is suitable for enhanced rosmarinic acid production; the selected optimized culture provide high-quality plant-derived products.

## 1. Introduction

While the global demand for plants for medical, cosmetological, and food use continues to increase, the plants themselves are subject to range restriction, uncontrolled overharvesting, and changing climatic conditions. Furthermore, due to increasing requirement, it is expected that the obtained plant raw material will be standardized and of high quality. As such, there is a need to identify alternative ways of obtaining plant resources [1].

One solution for the mass production of the valuable phytochemicals of economic interest could be based on plant biotechnology, as such approaches tend to be more environmentally friendly and efficient [2]. In vitro cultures employ controlled conditions to ensure standardized continuous production of plant cells and organs independent of plant life cycle, season, and climatic zones [2,3]. They do not require the use of pesticides or fertilizers, and they facilitate rapid multiplication of rare plant genotypes and the production of disease-free plants [3]. By careful selection of the obtained lines, it is possible to obtain efficient variants in a short time. Furthermore, production can be additionally increased by elicitation through external triggers such as physical and chemical stress; this has proved one of the most effective strategies for overproducing plant-derived metabolites of important commercial value [1,2,3]. Elicitors induce multiple physiological events, which increase the activation of a cascade of reactions such as expression of defence-related genes and accumulation of plant phytochemicals.

One physical factor which could be effective in regulating plant metabolic pathways is light. Light quality and quantity directly influence plant growth and chemical composition; therefore, light can be used as a convenient and highly modifiable factor to obtain vegetal material with tailored composition to specific applications. Such modulation is particularly effective on cell cultures such as calli, due to their homogeneous structure. Despite numerous studies on callus production, few studies have focused on the utilization of specific light wavelengths to optimize the production of biologically active compounds in callus cultures, especially in the context of medicinal plants. The effects of LED illumination in callus cultivation have been investigated in *Fagonia indica* Burm. f., *Lepidium sativum* L., and *Artemisia absinthium* L. [4,5,6]. The effect of light wavelength on secondary metabolite production in callus tissue was also evaluated in the case of two Lamiaceae species: *Ocimum basilicum* L. and *Ajuga bracteosa* Wall. ex Benth. [7,8]. Although such data are scarce, they do nonetheless indicate that the wavelength of LEDs may significantly modify the growth and production of bioactive compounds. However, further research is needed to establish the relationship between the type of wavelengths and secondary metabolism in plants grown in vitro. Currently, the mechanisms involved in these reactions are poorly understood, and as such, the optimal lighting conditions for a given culture are usually selected experimentally based on the plant species, the specificity of a given breeding line, and the type of metabolite.

In recent years, research on plant raw materials has focused on species that may be important in the treatment of lifestyle diseases, such as *Salvia* sp. belonging to the Lamiaceae family. This genus, which has over 1000 species, is widely used in medicine and cosmetology and as a food additive. These plants contain high levels of phenolic compounds with antioxidant and anti-inflammatory activity, i.e., substances that are able to neutralize free radicals and inhibit aging and cancer formation. One of the species is *Salvia atropatana* Bunge, which occurs in the natural environment in Iran, Turkey, Iraq, and Turkmenistan. In Iranian folk medicine, its shoots are used to treat infectious diseases, inflammations, and spastic conditions, and are used as an astringent and disinfectant [9]. In traditional Iranian herbal medicine, they were also used to treat digestive system diseases and diabetes [10]. Multiple classes of secondary metabolites, such as essential oil, phenolic acids, flavonoids, diterpenoids, and triterpenoids, have been isolated from the aerial parts and roots of *S. atropatana* [11,12,13,14,15].

The aim of the present research was to obtain an *S. atropatana* callus culture with high polyphenol production potential. This research evaluated the callogenesis response of in vitro-derived cotyledon, root, and hypocotyl explants to cytokinin supplementation. One of the key innovations of this article was that it employed LEDs emitting white (W), blue (B), red (R), and mixed 70% red and 30% blue light (R/B) to enhance biomass accumulation and secondary metabolite production in the sage callus culture. Apart from this, antioxidant potential of this selected optimized callus culture was investigated using ABTS (2,2′-azino-bis(3-ethylbenzothiazoline-6-sulfonic acid), DPPH (2,2-diphenyl-1-picrylhydrazyl), and FRAP (ferric reducing antioxidant power) assays. This is the first report on the use of biotechnological tools to obtain high-yielding crops of *S. atropatana*.

## 2. Results and Discussion

### 2.1. Optimization Callus Induction

*S. atropatana* in vitro culture was initiated from seeds; of these, 75% successfully germinated. The callus was observed on the explants between days 7 and 18 of cultivation. Typically, callus tissue began to form on the cutting site of the explant. All variants used turned out to be suitable for obtaining callus tissue, but the induction rate, the intensity of callus formation, and callus morphology varied according to the type of cytokinin in the medium and the type of explant used. The formation potential and morphological aspects of the resulting callus according to medium and explant type are shown in Table 1.

The maximum percent response of callus induction (100%) was observed for media containing cytokinins such as thidiazuron (TDZ, 1-phenyl-3-(1,2,3-thidiazol-5-yl)urea) and CPPU. In this case, callus formed on all explants regardless of their type (Table 1). After four weeks, callus tissue covered the entire explants from the hypocotyl and root fragments, but only part of the explants, in around half the cases, from the cotyledons. The initially obtained callus tissues were green or green-beige and friable except the one formed on the cotyledon in the presence of TDZ.

When benzylaminopurine (BAP) and meta-topoline (*m*-T) were present in the medium, the percentage of callus-forming explants ranged from 67–100%. However, callus covered practically the entire explants only for the cotyledon explants on *m*-T medium; in the remaining cases, callus growth varied. Moreover, even if the forming tissue was initially greenish or beige-greenish, darkening often occurred during the subculture. The medium supplemented with BAP turned out to be the least suitable for the formation of callus tissue on cotyledons; under these conditions, more than half of the explants formed a callus, but it formed only on the edges of the cotyledon and was dark brown and compact. Due to this lower potential for callus induction, and their compact nature and poor growth, these callus variants were eliminated from further cultivation.

Explant type and growth regulator combinations also played essential roles in improving the callus induction protocol of *Lamprocapnos spectabilis* (L.) Fukuhara [16] or *Ocimum sanctum* L., whose leaf explants showed higher callus formation potential than stem and inflorescence explants [17]. Although no studies have examined callus induction in *S. atropatana*, callus formation protocols have been reported for several other *Salvia* species. Stem explants from *Salvia miltiorrhiza* Bunge demonstrated higher potential for callus formation than leaves [18]. Callus tissue was induced in *S. fruticosa* Mill. by culturing leaves on MS medium containing TDZ [19], and in *S. officinalis* L. hypocotyls on MS medium with NAA, benzyladenine (BA) and 2,4-dichlorophenoxyacetic acid (2,4-D) [20]. Moreover, undifferentiated hypocotyl-derived *S. viridis* L. callus formed on both SH and MS medium with NAA, BA, and 2,4-D [21], and the most effective callus induction of *S. nemorosa* L. was achieved on medium supplemented with 2,4-D and BA [22].

Callus induction and growth requires exogenous application of cytokinin and auxin, although the type of growth regulators and their content must be adapted to the plant species. NAA was used in the present experiment; although 2,4-D is often used to obtain friable callus tissue, it may inhibit the biosynthesis of RA and other secondary metabolites, unlike NAA [23]. Also, some papers have reported NAA to have a beneficial effect on callus formation [16,20].

**Figure 1:**
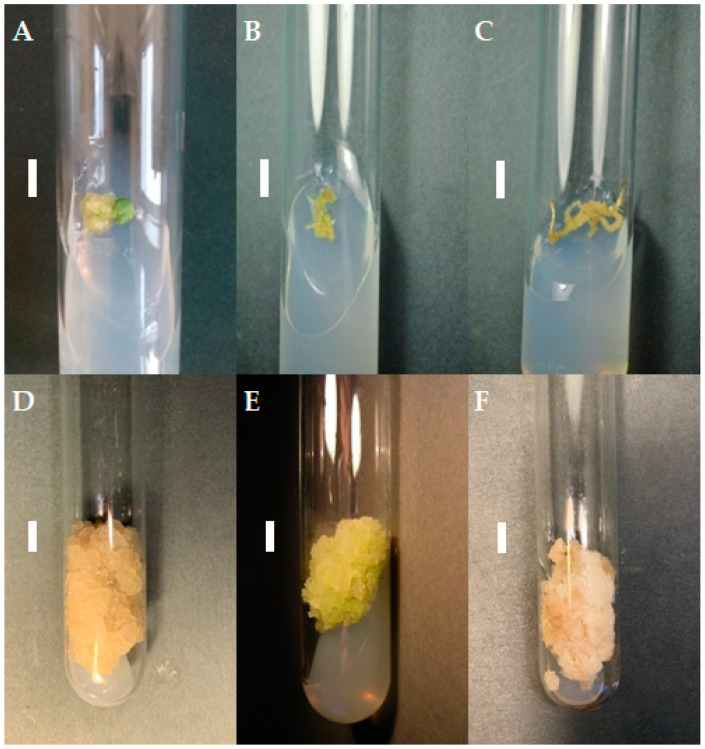
Callus tissue on MS medium supplemented with 0.2 mg/L NAA and 2 mg/L CPPU; the initial subculture (subculture 0): callus from cotyledon (**A**), hypocotyl (**B**), and root (**C**) fragments; subculture 20: cotyledon- (**D**), hypocotyl- (**E**), and root- (**F**) derived line. The duration of the subculture was four weeks. Bar = 1 cm.

If friable, viable callus tissue was noted on the *S. atropatana* explants after four weeks, it was transferred to a fresh medium with the same composition as that used for its initiation. If the callus remained viable, it was transferred onto individual medium variants for three subsequent four-week subcultures, and the growth of the callus and its morphology were noted (Table 1). The differences between individual callus lines became increasingly visible over subsequent subcultures. Some callus lines demonstrated aging: the cultures turned dark, stopped growing, and died. In the case of media supplemented with purine derivatives, *viz*. BAP and *m*-T, significant changes on callus color and morphology occurred during the first three subcultures, highlighted by the appearance of brown necrotic tissue. Callus tissue growth was limited. For the TDZ variants, callus of root and hypocotyl origin showed relatively intensive growth after the first transfer to fresh medium (subculture 1); however, this was significantly inhibited in subsequent passages and callus tissue adopted a more compact texture. In contrast, callus cultivated on CPPU-supplemented medium showed intensive growth in the subsequent subcultures regardless of the explant from which it was initiated (Table 1). Therefore, the following three independent lines grown on medium with CPPU were selected for further cultivation: a cotyledon-derived line (CL), a hypocotyl-derived line (HL), and a root-derived line (RL) (Figure 1).

### 2.2. Accumulation of Callus Biomass and Polyphenols in Callus Tissue

Culture of preselected *S. atropatana* callus lines (CL, HL, RL) was transferred every four weeks on fresh MS medium with 0.2 mg/L NAA and 2 mg/L CPPU. After about a year and a half, when the cultures demonstrated stable growth, their biomass accumulation and biosynthetic potential were estimated.

#### 2.2.1. Callus Growth

**Figure 2:**
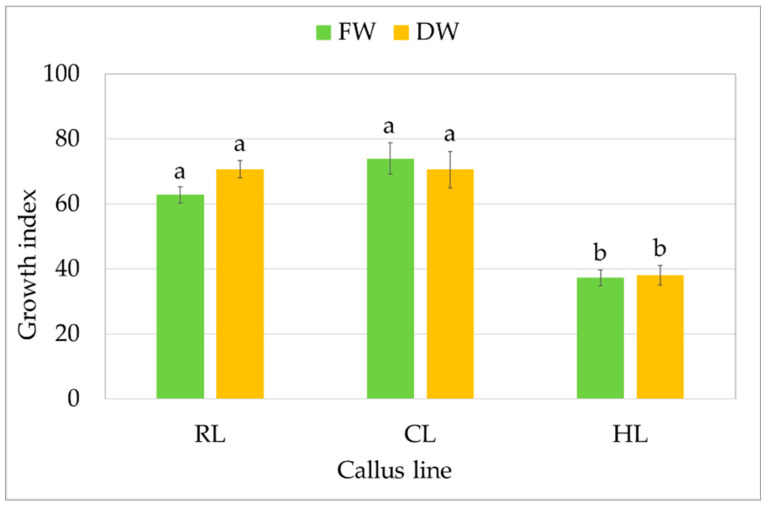
Growth of callus *S. atropatana*: RL (root line), CL (cotyledon line), and HL (hypocotyl) expressed as growth indices (GIs) of FW (fresh weight) and DW (dry weight). The values represent the mean ± standard error of three independent experiments. Means marked with the same letter for the same parameter were not significantly different (*p* < 0.05).

All three callus lines showed intensive growth on the medium on which they were originally initiated (Figure 1). CL and RL were creamy beige, and HL was light greenish; although all demonstrated a rather fragile texture, the RL callus was the loosest and driest. After a four-week growth period, fresh and dry weight were determined to compare the growth of callus lines. For the CL callus, the GI was 74 for FW, i.e., 9.03 ± 0.58 g per tube, and 71 for DW (0.35 ± 0.03 g per tube) (Figure 2). Similar GIs were found for RL, with 63 for FW and 71 for DW: no significant differences. The HL line showed significantly lower biomass accumulation, but with high GI values, reaching 40 within four weeks.

Previous studies have noted significant differences in callus growth according to explant type. In *Hypericum triquetrifolium* Turra, the most intense growth was found in the case of callus obtained from the stem, and the least from the roots [24]. In *Ocimum basilicum* and *Melissa officinalis* L., the best growth was obtained for the leaf-derived callus and the worst for the root [25]. These results confirm that each callus clone is a separate line with individual properties, growth, and biosynthetic potential.

#### 2.2.2. Polyphenol Accumulation

**Figure 3:**
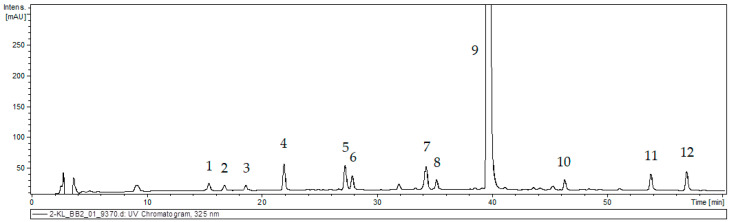
UPLC chromatogram of the extract of *S. atropatana* callus. The peak numbers are indicated in Table 2.

The 80% methanolic extract from callus tissue of *S. atropatana* was subjected to UPLC–MS analysis based on retention time (R_t_), UV, and mass spectra in the negative ion mode; the findings allowed a tentative identification of 12 phenolic acid derivatives (Figure 3, Table 2). The results were compared with those obtained for authentic standards and literature data [21,26,27,28].

The first group of metabolites was caffeic acid (CA, peak **4**), which exhibited a pseudomolecular [M − H]^−^ ion at *m*/*z* 179, and its three hexosides (CAH I, II, III; peak **1**, **2** and **3**) with a pseudomolecular ion [M − H]^−^ at *m*/*z* 341. These compounds are commonly detected in field-grown and in vitro *Salvia* species [21,28,29,30]. Compounds **5** and **6** were characterized as isomers of prolithospermic acid I and II (PRO I and II). They exhibited a pseudomolecular ion [M − H]^−^ at *m*/*z* 357 and fragmentation ions at *m*/*z* 313, indicating loss of CO_2_ and at *m*/*z* 203 corresponding to the neutral loss of 3,4-dihydroxylphenyl. A compound with the same fragmentation pattern has earlier been detected in roots of *S. miltiorrhiza* [27] and *S. viridis* [21]. Rosmarinic acid derivatives were also found in *S. atropatana* callus. Peak **9** was identified as RA. It exhibited pseudomolecular ion [M − H]^−^ at *m*/*z* 359 and fragmentation ions at *m*/*z* 197, 179 and161 corresponding with two molecular parts constituting this compound, i.e., danshensu and caffeic acid, and its dehydrated ion fragment. RA has earlier been detected in the aerial parts and roots of several *Salvia* species and in the roots and aerial parts of intact *S. atropatana* plant [12,26,28,29,30]. Peaks **7** and **8**, both exhibited pseudomolecular ion [M − H]^−^ at *m*/*z* 521, and yielded a fragment at *m*/*z* 359 (rosmarinic acid) from the neutral loss of the hexoside moiety. These were identified as rosmarinic acid hexoside I and II (RAH I and II). Peak **10** with *m*/*z* at 373 [M − H]^−^ and fragmentation peaks at *m*/*z* 179, 161 and 135 was identified as methyl rosmarinate (MRA). Compounds with analogous characteristics have been previously described for other sage species, also from in vitro culture [21,28,30,31]. Peaks **11** and **12** exhibited a [M − H]^−^ ion at *m*/*z* 313 and yielded fragment ions at *m*/*z* 269 and 161 corresponding to losses of CO_2_ and dihydroxybenzene units. They were identified as salvianolic acid F I and II (SAF I and II) isomers. Such structures have been previously found in shoots and roots of other *Salvia* species such as *S. bulleyana* [26], *S. viridis* [21], *S. euphratica* Montbret & Aucher ex Benth. and *S. verticillata* L [28].

All identified compounds were detected in all tested calli, but their content varied between individual lines (Table 3). The HPLC analysis found the predominant compound in hydromethanolic extracts of *S. atropatana* callus to be rosmarinic acid. It accounted for over 90% of the total polyphenol content. The highest RA content (27.5 mg/g DW) was found in CL callus; the amount was five times lower in RL callus, and even lower levels were found for HL callus (4.8 mg/g DW). On the other hand, both less-productive lines produced significantly higher RA content within four weeks than reported for the roots of several-years-old wild-grown *S. atropatana* plants (1.64 ± 0.24 mg/g DW) and only slightly less than in the leaves of these plants (6.55 ± 0.51 mg/g DW).

**Figure 4:**
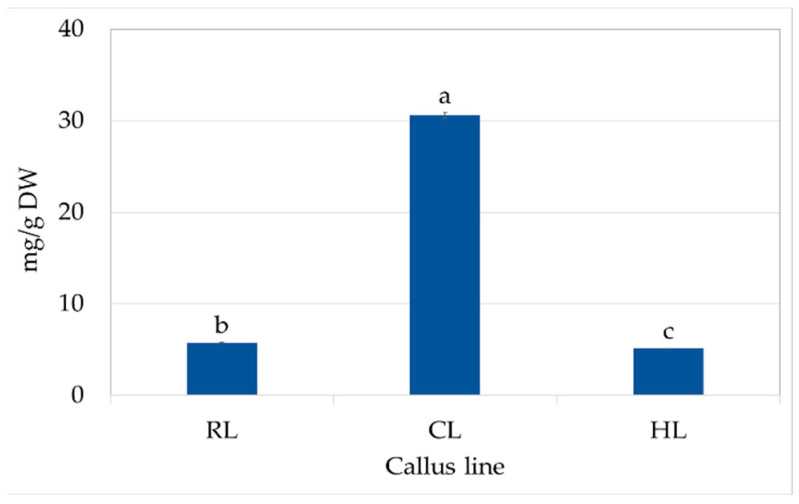
Total phenolic content in RL (root line), CL (cotyledon line), and HL (hypocotyl) callus cultivated on MS medium with 0.2 mg/L NAA and 2 mg/L CPPU after four weeks (subculture 18–20). The values represent the mean ± standard error of three independent experiments. Means marked with the same letter for the same parameter were not significantly different (*p* < 0.05).

CL callus also had high content of SAF I (0.61 mg/g DW), SAF II (0.62 mg/g DW), and CA (0.52 mg/g DW) (Table 3). These compounds were present at much lower levels in the callus of other lines, and only trace amounts of SAF isomers were found in the RL callus. Finally, the total polyphenol content in CL was five to six times higher than in the other callus lines (Figure 4).

Our findings indicate that the productivity of the *S. atropatana* callus line may be influenced by the type of explant from which the culture was initiated. This is in agreement with previous studies. Tarrahi and Rezanejad [32] report a higher anthocyanin level in leaf- and stem-derived calli of Rosa, compared with calli obtained from flowers, whereas Kulus and Tymoszuk [16] note that the calli of *Lamprocapnos spectabilis* derived from petioles and internodes contained more polyphenols than those obtained from leaves. The concentration of verbascoside has also been found to range from 4.3 mg/g DW in callus of a leaf-derived line of *Plantago ovata* Forssk. to 9.6 mg/g DW in a root-derived line [33]. Since the secondary metabolite accumulation in plants is genotype-specific, the selection of high-producing culture lines is an important step in obtaining valuable material which could represent a beneficial source of metabolites for commercial application [3].

Rosmarinic acid, the predominant polyphenol in *S. atropatana* callus, is well known to have a variety of pharmacological potential: anti-inflammatory, anti-allergic, antibacterial, antiviral, and cancer chemoprevention activities [34]. In addition, due to its high antioxidant capacity, it has possible application as a nutraceutical compound in the food industry and a valuable product for the cosmetics industry.

Although RA is commonly found in plants from the Lamiaceae family, including sage species, it is typically found at quite low levels, which are insufficient for commercial use [12]. In addition, its content can vary according to the location of plants, vegetation season, and climatic conditions [35]. Therefore, some reports have examined the possibility of RA accumulation in sage callus cultures. RA accumulated to a concentration of 21 mg/g dry weight in *S. fruticosa* leaf callus [19], and 15.8 mg/g in *S. officinalis* hypocotyl callus [20]. RA content was found to range from 3.6 to 11.3 mg/g DW in a 1.5-year-old callus of *S. viridis* depending on the callus line [21]. The study on *S. miltiorrhiza* also found that the origin of the callus influenced RA accumulation; callus initiated from the stem produced 4.5 times more RA (12.7 mg/g DW) than callus from the leaf (2.8 mg/g DW) [18], while relatively low RA content was noted in *S. nemorosa* callus culture (1.5 mg/g DW) [22].

It can be seen that the most efficient callus line of *S. atropatana* studied herein accumulated significantly greater amounts of RA. Therefore, this callus line (CL) was selected for further experiments.

### 2.3. Effect of Lighting Treatment on Callus Growth and Polyphenol Accumulation

In the next step, the lighting conditions were modified to stimulate secondary metabolism. This is a promising elicitation method in modern biotechnology [36]. Light has a fundamental role in plant physiological and biochemical processes and can promote or prohibit plant development and the secondary metabolite biosynthesis. In recent years, LED lighting with selected wavelengths has been successfully used in plant breeding, including in vitro plant cultures. By selecting the optimal lighting conditions for a given species, it is possible to obtain considerable increases in biomass and metabolite accumulation associated with medicinal properties [36]. Most existing reports have examined the influence of lighting conditions on the production of bioactive compounds in shoot and root cultures, and relatively few have examined callus cultures. LED treatment has been used to cultivate callus from several species, such as *Ocimum basilicum* [37], *Ajuga bracteosa* [8], *Lepidium sativum* [5], and *Artemisia absinthium* [6].

**Figure 5:**
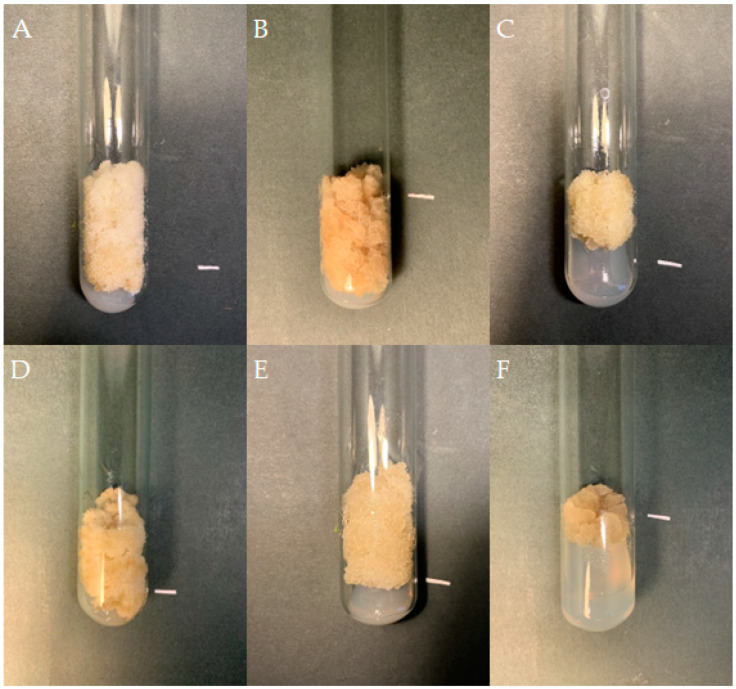
Callus tissue of *S. atropatana* cultivated on MS medium supplemented with 0.2 mg/L NAA and 2 mg/L CPPU under W (white) (**A**), R (red) (**B**), B (blue) (**C**), R/B (70% red and 30% blue) (**D**) LEDs, FL (fluorescent lamps) (**E**), and in D (dark) (**F**) (subculture 33). The duration of the subculture was four weeks. Bar = 1 cm.

**Figure 6:**
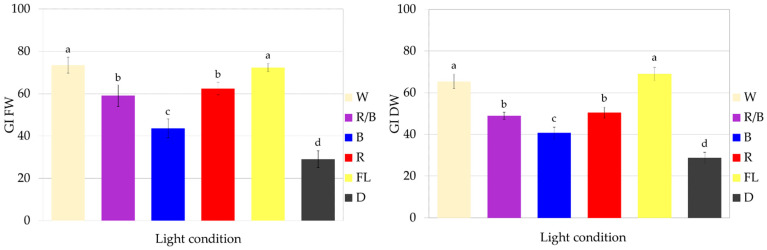
Growth of *S. atropatana* CL (cotyledon line) callus under different light conditions expressed as growth indices (GIs) of FW (fresh weight) and DW (dry weight). The duration of the subculture was four weeks. The values represent the mean ± standard error of three independent experiments. Means marked with the same letter for the same parameter were not significantly different (*p* < 0.05).

The optimal lighting conditions for the growth of fresh and dry biomass of CL callus culture of *S. atropatana* were found to involve full-spectrum white lighting, from both fluorescent and LED sources. Such exposure resulted in FW of 8.82–8.96 g and DW 0.31–0.35 g within four weeks, corresponding to 72–73 FW GI and 64–69 DW GI (Figure 5 and Figure 6).

Although exposure to red and mixed LEDs also strongly stimulated culture growth, they were less effective than treatment with white light. These conditions resulted in approximately 60 FW GI and 50 DW GI (Figure 6). The lowest CL callus growth was observed in the dark (Figure 6), with FW and DW values being approximately 2.5 times lower than those reported under optimal treatment.

The color and consistency of callus tissue differed according to lighting condition (Figure 5). Callus was noticeably darker with light orange fragments under red light, darker with a dirty beige shade when grown in the dark, and very bright and loose under white LEDs.

**Figure 7:**
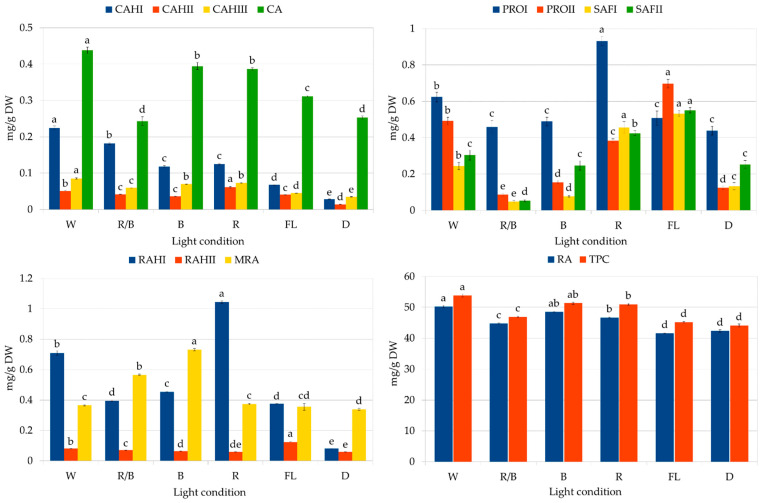
Polyphenol content in CL (cotyledon line) callus of *S. atropatana* cultivated on MS medium supplemented with 0.2 mg/L NAA and 2 mg/L CPPU under W (white), R (red), B (blue), R/B (70% red and 30% blue) LEDs, FL (fluorescent lamps), and in D (dark) after four weeks. CA—caffeic acid; CAH I, CAH II, CAH III—caffeic acid hexoside I, II, III; RAH I, RAH II—rosmarinic acid hexoside I, II; MRA—methyl rosmarinate; PRO I, PRO II—prolithospermic acid I, II; SAF I and II—salvianolic acid F isomers I and II; RA—rosmarinic acid; TPC—total polyphenol content. The values represent the mean ± standard error of three independent experiments. Means marked with the same letter for the same parameter were not significantly different (*p* < 0.05).

The second part of the study assessed the influence of lighting conditions on the production of polyphenolic compounds in callus. The highest RA content was noted in the callus grown under white LEDs (Figure 7): 50.25 mg/g DW, followed by blue light (48.53 mg/g DW). The lowest level was found in callus grown in the dark and under fluorescent lamps (41.6 to 42.4 mg/g DW). It can be seen that by optimizing the lighting conditions, it was possible to increase the RA content by 20%.

Exposure of callus to white LEDs increased the biosynthesis of most analyzed polyphenolic compounds, resulting in the highest total phenolic content (TPC) (53.9 mg/g DW). In contrast, white fluorescent light treatment resulted in the lowest TPC value, although the greatest accumulation of salvianolic acid F isomers (Figure 7); this indicates the stimulation of different biosynthetic pathways in these conditions. However, it can be seen that the levels of RA and total phenols in CL callus grown under fluorescent lamps increased by 75% and 50%, respectively, over a year (Figure 4 and Figure 7, Table 3). Similarly, the RA content in *S. officinalis* callus increased by 40% over a year, reaching a value of 15.8 mg/g DW [20], while its content in *S. viridis* doubled between passages 12 and 18 [21]. An increase in the production of iridoids and isoverbascoside was also described for *Harpagophytum procumbens* (Burch.) DC. ex Meisn. callus tissues between 1.5 and 2.5 years of culture [38]; however, in this case, the level of verbascoside remained constant throughout the entire study period.

Only a few reports have examined the influence of light application on the growth and production of secondary metabolites in sage species, concerning shoots and transformed roots of *S. miltiorrhiza* and *S. bulleyana* Diels [39,40,41,42]. For both species, optimal conditions for biomass and metabolite accumulation were obtained for shoots grown under mixed red/blue LEDs with a clear dominance of red light [41,42]. A mixture of blue and red light also stimulated RA biosynthesis in hairy roots of *S. miltiorrhiza* in comparison to dark conditions [39]. In contrast, LED treatments inhibited the growth and production of polyphenols in transformed *S. bulleyana* roots, regardless of the spectrum used [40].

Our report is the first to examine the effect of LED treatment on *Salvia* sp. callus culture. Only two earlier reports have described the production of RA in callus culture, both of which concern *Ocimum basilicum* [7,37]. They found that the varieties demonstrate significantly different levels of RA biosynthesis in response to lighting conditions: of seven light treatments, the highest level of RA was observed under blue light for *O. basilicum* [7], and in the dark for *O. basilicum var. purpurascens* [37]. In our studies, although darkness was not optimal for RA production, it did not reduce its level dramatically, confirming that light is not necessary for RA biosynthesis; however, the lack of light had a negative effect on the growth of *S. atropatana* callus.

Several other reports have examined the production of other phenolic acids or TPC in callus tissue. Similar to our results, it was found that *Lepidium sativum* and *Fagonia indica* accumulate the highest levels of polyphenolic acids in callus growing under white LEDs [4,5]. However, *Ajuga bracteosa* callus demonstrated optimal polyphenol biosynthesis under blue light [8]. In the present study, *S. atropatana* callus accumulated similar RA content and TPC under blue and white LEDs.

Some evidence suggests that exposure to LEDs, especially blue LEDs, may photoinduce phenolic synthesis [36]. Blue light would stimulate cytochrome P450, resulting in elevated reactive oxygen species (ROS) levels; as a result, polyphenol production may be increased to protect against the generated ROS [43]. Indeed, blue LED treatment was found to stimulate flavonoid biosynthesis in *Dracocephalum forrestii* shoots, and its level correlated with strong oxidative stress, reflected in high levels of antioxidant enzymes [44]. Blue light was also found to induce the highest level of stress in *S. bulleyana* shoots, but such exposure was not optimal for either growth or polyphenolic compound production in the culture; the authors propose that in this case, the stress may have been too high, which limited the growth and development of shoots [41]. Also, in *F. indica*, white LED treatment was found to induce optimal stress for achieving the highest levels of flavonoids and caffeic acid, although higher antioxidant enzyme activity was recorded under blue LEDs [45]. On the other hand, all LED treatments induced greater antioxidant enzyme activity and higher RA levels in *Dracocephalum forrestii* shoots compared to fluorescent light [44]. Similar results were obtained in the present study for callus culture of *S. atropatana*: the lowest RA production was associated with white fluorescent lamp treatment.

Our present findings, and most of the above studies, indicate that light with a wider spectrum yields more positive effects on biosynthetic potential of callus tissue than only monochromatic blue or red LEDs. This could be due to the synergistic effects of combined wavelengths, which are able to stimulate several different photoreceptors simultaneously, i.e., phytochrome receptors for red light, and cryptochromes and phototropins for blue light; this in turn upregulates the metabolic pathways necessary for the synthesis of bioactive compounds in plants. In contrast, Chen et al. [46] found combined red and blue LEDs to have an antagonistic effect on the metabolic pathways of some phytochemicals in potato. In the present study, combined blue and red light treatment did not yield strong metabolism stimulation in *S. atropatana* callus. Other studies have shown that the plant response can be significantly modified by altering the ratios between blue and red LEDs [39], which suggests the need for further research in this area. It is also possible that additional wavelengths other than red emitted by white LEDs might enhance the effects of blue light on the metabolism of *S. atropatana*.

### 2.4. In Vitro Antioxidant Potential of Callus Culture

Stress conditions during in vitro cultivation can stimulate polyphenol production, including phenolic acids like rosmarinic acid, in plant material, which may enhance its antioxidant potential [47]. Hence, this study also assessed the biological activity of highly productive *S. atropatana* culture.

Several factors influence the antioxidant activity of callus tissue, such as plant genotype and culture conditions. Optimizing these factors can boost the production of antioxidant compounds in callus cultures, thus improving their antioxidant potential. Given that the optimized *S. atropatana* callus culture grown under white LEDs yielded much higher levels of rosmarinic acid compared to naturally grown *Salvia* species [12], it is unsurprising that it also exhibited strong scavenging potential against free radicals (Table 4).

Antioxidant assays, measuring the ability of antioxidants to scavenge free radicals, indicate their capacity to inhibit oxidative processes. The IC_50_ (half-maximal inhibitory concentration) value of the examined extract was 16.0 µg/mL for ABTS and 26.9 µg/mL for the DPPH assay, similar to those of the reference antioxidant butylated hydroxytoluene (BHT). Research on the antiradical properties of *Salvia* species is of considerable interest. For instance, IC_50_ values for the ABTS test for various sages ranged from 10–70 µg/mL [30,48], while for the DPPH assay, they varied from 2–500 µg/mL, with most falling between 20 and 50 µg/mL [48,49]. Due to the lower rosmarinic acid content in the field-grown *S. atropatana* plants, their IC_50_ value for DPPH assay was significantly higher (69 µg/mL) [12,49] than that of the callus culture extract. On the other hand, the antioxidant activity of intact sages may also be attributed to other secondary metabolites such as flavonoids, diterpenoids, or triterpenoids, which are present in field-grown plants but were not detected in the analyzed callus culture.

Studies on the antiradical activity of callus tissue, which could be comparable to our results, are scarce, especially within the genus *Salvia*. Notably, *Satureja hortensis* L. calli exhibited strong anti-DPPH activity, comparable to our findings [50]. However, most of the tested callus cultures, like *Salvia officinalis* L. (81.7 µg/mL) [51], *Origanum acutidens* (Hand.-Mazz.) Ietsw. (71.5 µg/mL) [52], and *Dracocephalum moldavica* L. (61.85 µg/mL) [53], demonstrated significantly lower activity due to their lower content of secondary metabolites, including rosmarinic acid.

The callus extract of *S. atropatana* also demonstrated good reducing ability for Fe^3+^ ions (1.79 mM Fe (II)/g DW of extract), similar to that reported for the aerial parts of intact plants of *S. viridis* and *S. bulleyana* [26,54]. Equally strong activity in FRAP assay was exhibited by the extract of *D. moldavica* suspension culture (1.62 mM Fe(II)/g DW), whereas the callus tissue of this species characterized by lower rosmarinic acid content showed significantly lower reduction ability (1.01 mM Fe(II)/g DW).

## 3. Materials and Methods

### 3.1. Culture Establishment

Cultures were established based on *S. atropatana* seeds obtained from the Kärntner Botanikzentrum (Klagenfurt am Wörthersee, Austria). The seeds were surface-sterilized for one minute with 70% (*v*/*v*) ethanol, followed by 2% (*v*/*v*) sodium hypochlorite for 10 min, and then they were washed three times with distilled water. The sterilized seeds were placed onto solidified MS [55] medium with 0.02 mg/L kinetin and 1 mg/L gibberellic acid for germination. The cultures were incubated at a temperature of 24 °C ± 2 °C in darkness.

### 3.2. Callus Induction and Preliminary Line Selection

Three-week-old seedlings of *S. atropatana* developed from the seeds, were divided into fragments (cotyledons, root, and hypocotyl fragments), and then transferred onto MS medium supplemented with 0.2 mg/L NAA and different cytokinins for callus induction: 2 mg/L BAP, 2 mg/L *m*-T, 2 mg/L CPPU, or 0.5 mg/L TDZ. The cultures were incubated at 24 ± 2 °C under a 16 h photoperiod at a light intensity of 35 µM/m^2^∙s provided by cool white fluorescent lamps (SMARTLUX PRO, 6500K, Osram, Germany). Medium compounds and all plant growth regulators were purchased from Duchefa Biochemie (BH Haarlem, The Netherlands).

After four weeks, the explants were monitored for percent callus induction and its morphology. Three replicates were performed per each treatment.

Friable, vital callus tissue was transferred after four weeks onto fresh hormonal medium for further cultivation in the same conditions. The growth of calli and their morphological parameters were recorded for another three four-week subcultures (S_1_–S_3_).

### 3.3. Growth of Selected Callus Lines

After three subcultures lasting four weeks each, three well-grown callus variants were selected for further experiments. One was generated from a cotyledon (CL line), another from a hypocotyl (HL), and another from a root (RL). All were cultured on MS medium with 0.2 mg/L NAA and 2 mg/L CPPU at 24 ± 2 °C under a 16 h photoperiod under white fluorescent lamps (photosynthetic photon flux density, PPFD = 35 µM/m^2^∙s) for subsequent passages until their growth became stable. Following this, the growth of the lines and their polyphenolic compound accumulation were compared.

The experiment was performed in triplicate. The callus tissues used in the experiment were derived from subcultures 18 to 20. The mean biomass of callus inoculum was *cir.* 0.13 g fresh weight, and 0.005 g dry weight. Callus tissues were harvested after 28 days of culture and the final FW was measured. Following this, the calli were frozen and lyophilized, and their DW was determined. The average growth index was calculated based on callus FW and DW using the formula: GI = [(Gf − Gi)/Gi], where Gf is the callus biomass at the end of the cultivation period and Gi is the initial biomass of the callus (biomass of inoculum).

The obtained plant material was then analyzed for the presence of polyphenolic compounds.

### 3.4. Qualitative and Quantitative Polyphenolic Compound Analysis

Samples of lyophilized and micronized callus tissue were extracted with 30 mL 80% methanol using an ultrasound bath (Techpan, Warsaw, Poland) at 40 °C for 15 min, and then repeating twice with 15 mL of the extraction solution. The combined extracts were evaporated to dryness under reduced pressure and stored at 4 °C until further analysis.

The polyphenolic compounds were subjected to qualitative analysis using a UPLC–DAD–ESI-MS/MS system consisting of a UPLC-3000 RS apparatus (Dionex, Dreieich, Germany) with DAD detection and an AmaZon SL ion trap mass spectrometer with an ESI interface (Bruker Daltonik GmbH, Bremen, Germany). Separation was performed on a Zorbax SB-C18 column (150 × 2.1 mm, 1.9 μm) (Agilent, Santa Clara, CA, USA) with a flow rate of 0.2 mL/min and a temperature of 25 °C. The mobile phase consisted of 0.1% HCOOH in water (A) and 0.1% HCOOH in acetonitrile (B) on a gradient of 5–40% B from 0 to 60 min. The LC eluate was introduced into the ESI interface without splitting, and compounds were analyzed in negative ion mode with the following settings: nebulizer pressure of 40 psi, drying gas flow rate of 9 L/min, nitrogen gas temperature of 300 °C, capillary voltage of 4.5 kV. The mass scan ranged from 100 to 2200 *m*/*z*. UV spectra were recorded in the range of 200–400 nm. Compounds were tentatively identified based on their retention time and UV and mass spectra by comparison with literature data [21,26,27,28].

Polyphenolic compound content was determined using an Agilent Technologies 1290 Infinity HPLC apparatus (Santa Clara, CA, USA) with DAD detector. The extracts were dissolved in 2 mL of 80% methanol and filtered into HPLC vials using a 0.22 µm nylon filter. The analysis was performed using an Eclipse XDB-C18 column (4.6 × 150 mm, 5 µm) with a flow rate of 1.6 mL/min and a temperature of 35 °C. The mobile phase consisted of acetonitrile (solvent A) and water with 0.1% formic acid (solvent B). A gradient elution was used as follows: 0–5 min 10–18% A; 5–20 min 18–38%A, 20–25 min, 38–100% A, 25–30 min, 100% A (isocratic elution), and 30–37 min 10% A (equilibration). The peaks were monitored at λ = 325 nm.

Calibration was performed using authentic standards of caffeic acid (CA), rosmarinic acid (RA), and salvianolic acid F (SAF) purchased from Chem Faces Biochemical Co., Ltd. (Wuhan, China). For the polyphenols without available authentic standards, quantification was based on the calibration curve of a similar compound: CA for CAH I, II and III, RA for isomers of PRO I, and II, RAH I, II, and MRA, SAF for isomers of SAF I and II. The injection volume was set to 10 µL. All analysis were performed in triplicate, and the results are presented as mg/g DW. The total phenolic content was calculated on the basis of the sum of the contents in a sample of all quantified polyphenols.

### 3.5. Effect of Lighting Treatment on Callus Growth and Polyphenol Accumulation

**Figure 8:**
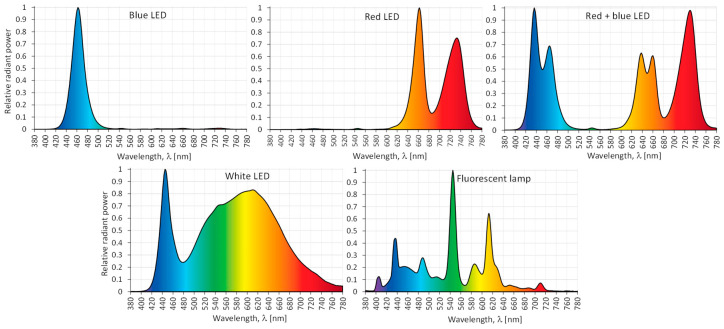
Relative spectral characteristics of the light emitted by the tested LEDs and fluorescent lamps.

The cotyledon-derived callus (CL) of *S. atropatana* was placed in special growth chambers (1 m × 0.68 m) equipped with different LED light combinations (PXM Sp., Niepołomice, Poland): under white (3900 K), blue (λ_max_ = 460 nm), red (λ_max_ = 660 and 730 nm), and mixed 70% red and 30% blue LEDs (λ_max_ = 435 nm, 460 nm, 635 nm, 660 nm and 730 nm), and under fluorescent lamps (Figure 8). Some cultures were maintained in the dark.

The mean PPFD for all light treatments was approximately 35 μM/m^2^∙s, with variations of no more than 10% from the mean, depending on the distance from the light source. PPFD values were determined based on the recorded spectral characteristics. Measurements were taken with a portable GL SPECTIS 1.0 Touch spectrometer (GL Optic, Puszczykowo, Poland). PPFD was determined by creating a measurement grid consisting of a set of evenly spaced points. The EN 12464-1 standard was used [56], allowing the maximum dimension of the grid to be determined according to the equation: $p=\;0.2\cdot 5^{\log_{10}d}$, where: d—longer dimension of the area [m] and p—the maximum grid cell size [m]. With these data, the dimensions of the individual meshes of the measurement grid were determined (0.20 m × 0.17 m). A total of 20 meshes were obtained. In the central place of each elementary field (grid mesh), the radiant power (spectral irradiance) of radiation emitted for each type of light source was measured.

In this part of study, the effects of the various light conditions on callus growth and polyphenol accumulation were determined. The experiment was performed in triplicate (subcultures 32–34). After 28 days of light treatment, the callus cultures were harvested to determine FW and DW and polyphenol level, as described above.

### 3.6. In Vitro Antioxidant Assays

The DPPH and ABTS assays were carried out according to the method described by Grzegorczyk-Karolak and Kiss [54] for seven final extract concentrations (1, 5, 10, 20, 50, 100, and 200 µg/mL). For DPPH test, the absorbance was recorded at 517 nm after a 30 min incubation period in darkness at room temperature, while for ABTS assay, at 734 nm after a 10 min incubation. The results for ABTS and DPPH tests were expressed as IC_50_ value which represents the concentration of the extract able to scavenge 50% of the initial radical concentration.

The ferric reducing ability of extract (FRAP assay) was evaluated following the procedure described by Grzegorczyk-Karolak and Kiss [54]. The absorbance of reaction mixture was measured at 595 nm after 15 min of incubation at 37 °C in darkness, and the results were expressed as µM Fe(II)/g of extract dry weight.

A Ray Leigh UV-1601 spectrophotometer (Beijing Reyleigh Corp., Beijing, China) was used for the absorbance measurement and BHT was used as a positive control in all antioxidant assays.

### 3.7. Data Analysis

Each experiment was replicated at least three times, and the results were expressed as mean ± standard error (SE). Comparisons were performed using ANOVA, followed by Tukey’s *post hoc* test. Relationships characterized by *p* < 0.05 were regarded as significant. The collected data were analyzed using Excel version 2013 (Microsoft Inc., Redmond, WA, USA), and STATISTICA 13 PL software (StatSoft, Krakow, Poland).

## 4. Conclusions

A callus line of *S. atropatana* with a high potential for polyphenol production was successfully obtained, indicating that it is possible to significantly modify the callogenesis response by the selection of appropriate explants and cytokinins. This also gives the first evidence that the use of LEDs could be a valuable tool in optimizing callus cultures for higher yields. In the present study, a combination of correct line selection and LED treatment promoted biomass accumulation while increasing the production of valuable polyphenols in *S. atropatana* callus. After optimizing the growth conditions, the biosynthesis of rosmarinic acid in the callus exceeded 50 mg/g DW during the four-week subculture, i.e., 7.5 times higher than that found in leaves and 30 times higher than that in the roots of several-year-old field growing plants. In addition, the optimized callus culture indicated strong antioxidant potential, especially radical scavenging ability. However, further research is needed to determine the molecular mechanism underlying the processes responsible for the production of secondary metabolites in sage callus tissue. These findings could facilitate metabolic engineering approaches aimed at enhancing the antioxidant properties of sage and related species.

## Figures and Tables

**Table 1:** The influence of plant growth regulator and explant on *S. atropatana* callus induction and its morphology.

Type of Cytokinin	Type of Explant	Callus Formation (S_0_)			Callus Growth (S_1_–S_3_)
		Percentage of Callus Formation	The Intensivity of Formation	Morphology	
BAP	Hypocotyl	73.3	50% +++ 50% +	Light green	Poor growth, gradually darkens from the inside
	Root	100	53.3% +++ 46.7% +	Green-beige, darkening at the edges	Poor growth, gradually darkens from the inside
	Cotyledon	66.7	100% +	Dark, formed only at the edges of the explant	-
*m*-T	Hypocotyl	78.6	100% ++	Beige, quick to darkens	Poor growth, darkens
	Root	100	33.3% +++ 66.7% ++	Soft, green, darkens at the edges	Poor growth, darkens
	Cotyledon	100	100% +++	Rich, green-beige	With subsequent subcultures grows less and darkens
TDZ	Hypocotyl	100	100% +++	Green-beige, quite friable	Moderate growth, darkens over time
	Root	100	100% +++	Green-beige, friable	Initially grows abundantly, but darkens in subsequent subcultures
	Cotyledon	100	66.7% ++ 33.3% +	Green, quite compact	Gradually darkened, grows quite poorly
CPPU	Hypocotyl	100	100% +++	Greenish, friable	Greenish and friable, grows rapidly
	Root	100	100% +++	Green-beige, friable	Green-beige and friable, grows rapidly
	Cotyledon	100	50% +++ 50% +	Green-beige, on some explants only at the cutting site	Green-beige, growing intensively

Callus formation intensity: +++ abundant, ++ moderate, + poor; S_0_, S_1_–S_3_: subculture 0, subculture 1–3.

**Table 2:** UPLC–MS data on polyphenolic compound identified in *S. atropatana* callus.

Peak No.	R_t_ [min]	[M − H]^−^	Main Fragments	Tentative Compound
**1**	15.4	341	281, 203, 179, 161, 135	Caffeic acid hexoside I
**2**	16.9	341	281, 251, 221,	Caffeic acid hexoside II
**3**	18.6	341	281, 251, 221, 179, 135	Caffeic acid hexoside III
**4**	21.9	179	135	Caffeic acid
**5**	27.3	357	313, 295, 269, 247, 203, 159	Prolithospermic acid I
**6**	27.8	357	313, 295, 269, 247, 203, 159	Prolithospermic acid II
**7**	34.1	521	359, 223, 197, 179, 161	Rosmarinic acid hexoside I
**8**	34.3	521	359, 223, 197, 179, 161	Rosmarinic acid hexoside II
**9**	39.7	359	223, 197, 179, 161	Rosmarinic acid
**10**	46.4	373	179, 161, 135	Methyl rosmarinate
**11**	53.8	313	269, 161	Salvianolic acid F isomer I
**12**	56.6	313	269, 203, 161	Salvianolic acid F isomer II

**Table 3:** Polyphenol content in CL (cotyledon line), HL (hypocotyl line), and RL (root line) callus cultivated on MS medium with 0.2 mg/L NAA and 2 mg/L CPPU after four weeks (subculture 18–20).

Compound [mg/g DW]	CL	HL	RL
CAH I	0.050 ± 0.0003 a	tr.	0.030 ± 0.0002 b
CAH II	0.046 ± 0.0004 a	tr.	tr.
CAH III	0.045 ± 0.0003 a	tr.	tr.
CA	0.522 ± 0.005 a	0.072 ± 0.001 b	0.044 ± 0.0004 c
PRO I	0.260 ± 0.004 a	0.032 ± 0.001 b	0.025 ± 0.001 c
PRO II	0.386 ± 0.005 a	tr.	tr.
RAH I	0.381 ± 0.003 a	0.035 ± 0.001 b	0.040 ± 0.002 b
RAH II	0.108 ± 0.001 a	tr.	tr.
RA	27.46 ± 0.22 a	4.83 ± 0.022 c	5.57 ± 0.033 b
MRA	0.198 ± 0.007 a	0.043 ± 0.003 b	0.042 ± 0.001 b
SAF I	0.555 ± 0.033 a	0.046 ± 0.006 b	tr.
SAF II	0.605 ± 0.007 a	0.070 ± 0.007 b	tr.

tr.—traces. The values represent the mean ± standard error of three independent experiments. Means marked with the same letter for the same parameter were not significantly different (*p* < 0.05).

**Table 4:** Antioxidant activities of extract of *S. atropatana* cotyledon line (CL) callus determined by the ABTS, DPPH, and FRAP assays.

Antioxidant Assay	CL	BHT
ABTS [IC_50_, μg/mL]	16.0 ± 0.52 b	9.86 ± 0.85 a
DPPH assay [IC_50_, μg/mL]	26.9 ± 0.06 a	26.40 ± 1.26 a
FRAP assay [mM Fe (II)/g DW]	1.79 ± 0.01 b	3.67 ± 0.27 a

The values represent the mean ± standard error of three independent experiments. Means marked with the same letter for the same parameter were not significantly different (*p* < 0.05).

## Data Availability

The original contributions presented in the study are included in the article, further inquiries can be directed to the corresponding authors.
